# Job vacancies at the European Centre for Disease Prevention and Control (ECDC)

**DOI:** 10.2807/1560-7917.ES.2022.27.50.2212151

**Published:** 2022-12-15

**Authors:** Eurosurveillance editorial team

**Affiliations:** 1 European Centre for Disease Prevention and Control (ECDC), Stockholm, Sweden

The European Centre for Disease Prevention and Control (ECDC) invites applications for the following positions:

 

**Expert Data Management**
The application deadline expires on 16 Jan 2023.

 

**Principal Expert Microbiology**
The application deadline expires on 20 Jan 2023.

 

For more information, please visit the ECDC website: https://www.ecdc.europa.eu/en/vacancies

